# The mosquito gut microbiome and *Plasmodium* development: a scoping review of molecular approaches and implications for malaria vector control

**DOI:** 10.3389/fcimb.2026.1906816

**Published:** 2026-09-30

**Authors:** Wisdom Deborah Cleanclay, Fabrice Banadzem Kernyuy, Irrinus Fonyuy Kintung, Nina Ghislaine Yensii, Agnes Mbiaya Mbeng Obi, Ornella Anaïse Mbang Nguema

**Affiliations:** 1 Department of Biochemistry, Covenant University, Ota, Ogun State, Nigeria; 2 Covenant University Public Health and Wellbeing Research Cluster (CUPHWERC), Covenant University, Ota, Ogun State, Nigeria; 3 Covenant Applied Informatics and Communication Africa Centre of Excellence (CApIC-ACE), Covenant University, Ota, Ogun State, Nigeria; 4 Department of Microbiology, Covenant University, Ota, Ogun State, Nigeria; 5 Biomedical Research Centre on Infectious Pathogens and Associated Pathologies (CREIPA), Department of Parasitology-Mycology and Tropical Medicine (DPMTM), University of Health Sciences, Owendo, Gabon; 6 Medical Entomology Unit, DPMTM, USS, Owendo, Gabon

**Keywords:** Plasmodium, 16S rRNA, Anopheles, malaria vector control, microbiome profiling, mosquito gut microbiome, paratransgenesis

## Abstract

**Background:**

Malaria remains a major health burden, with an estimated 282 million cases and approximately 610,000 deaths recorded in 2024. The increasing prevalence of insecticide-resistant *Anopheles* vectors and drug-resistant *Plasmodium* strains has substantially eroded the efficacy of conventional control strategies, underscoring the critical need for innovative approaches. Accumulating evidence suggest that the mosquito midgut microbiome exerts a significant influence on *Plasmodium sporogonic* development; however, no scoping investigation has fully mapped the metagenomic evidence base in this field.

**Methods:**

Adhering to the Arksey and O’Malley framework and PRISMA-ScR guidelines, a systematic search was conducted across PubMed, Scopus, and Web of Science for publications from January 2000 to April 2026. Studies that used microbiological, molecular, or metagenomic techniques to study the gut microbiota of mosquito species related to *Plasmodium* were included. Descriptive mapping was used to synthesize the data in a thematic manner.

**Results:**

Thirteen primary studies met the inclusion criteria, comprising both observational field surveys and experimental infection studies. Four themes emerged: (1) gut microbiota diversity and composition are associated with *Plasmodium* infection status, though the direction varies by mosquito species, geography, and season; (2) several taxa, including *Burkholderia*, *Asaia*, *Sphingomonas*, *Pseudomonas*, and *Serratia*, are consistently associated with reduced infection or are experimentally inhibitory; (3) the *Enterobacteriaceae* and *Serratia* spp. show context-dependent, bidirectional relationships with *Plasmodium* that are best explained by strain-level variation; and (4) a smaller set of taxa, including *Elizabethkingia*, *Chryseobacterium*, *Methylobacterium*, and *Pantoea*, is positively associated with infection, on observational evidence only. The evidence relied predominantly on 16S rRNA amplicon sequencing, with a minority of studies adding whole-genome sequencing, transcriptomics, or metabolomics; none used shotgun metagenomics.

**Conclusions:**

The mosquito gut microbiome exerts a net inhibitory effect on *Plasmodium* development, with translational potential for microbiome-based vector control, including paratransgenesis. However, the microbiota is not uniformly antagonistic to the parasite, and taxa positively associated with infection remain observational. The field is limited by a near-exclusive reliance on 16S rRNA profiling, few functional and longitudinal studies, and geographically narrow evidence. Multi-omics approaches, longitudinal designs, and broader geographic coverage will be essential to translate these associations into sustainable malaria control.

## Introduction

Malaria remains a life-threatening disease with profound effects on the human genome, as demonstrated by the positive selection of protective genetic traits such as the sickle cell trait and beta-thalassemia (Chinedu et al., 2024; Cleanclay et al., 2026; Wakai et al., 2026). In sub-Saharan Africa, malaria remains a major public health concern, affecting more than 90% of the population and contributing to high levels of morbidity and mortality, despite ongoing global control efforts (Adebayo et al., 2018; Oladipo et al., 2022). The number of malaria cases reached 282 million, and approximately 610,000 deaths in 2024, with transmission occurring in about 80 malaria-endemic countries worldwide (World Malaria Report, 2025).

Malaria is a life-threatening infectious disease caused by *Plasmodium* parasites, which are transmitted to humans through the bites of infected female *Anopheles* mosquitoes (Adebayo et al., 2018; Su et al., 2020). The most common species of *Plasmodium* that infect humans are *Plasmodium malariae*, *Plasmodium vivax*, *Plasmodium ovale*, *Plasmodium falciparum*, and *Plasmodium knowlesi*, with each having varying severity in different geographical areas (Naandam et al., 2025). *P. falciparum* is the most fatal of these, and it grows exponentially in the bloodstream and mostly infects African children, causing severe anemia and the highest numbers of deaths related to malaria (Nwinyi et al., [[NoYear]]; Varo et al., 2020). Malaria is one of the major causes of deaths, particularly among children under 5 years of age and though a variety of preventive interventions, such as chemoprevention for pregnant women and infants, insecticide-treated bed nets, vaccination and indoor residual spraying, are now in use in sub-Saharan Africa, the disease still poses a significant threat to the health of the region’s inhabitants (Tebamifor et al., 2025; Wakai et al., 2025). Resistance to drugs and insecticides in both *Plasmodium* strains and *Anopheles* vectors has been a serious challenge to the effectiveness of traditional control methods (Cleanclay et al., 2026; Yensii et al., 2026). This changing resistance landscape, exacerbated by the dearth of entomological monitoring data, increasing vector habitat from climate change, and low malaria vaccination rates, underscores the pressing need for new, environmentally friendly strategies to control malaria (Charavanamuttu et al., 2026).

The bacteria, fungi, viruses and other microorganisms make up the mosquito midgut. This community is in dynamic interaction with invading pathogen and the mosquito host (Liu et al., 2024). The role of the microbial communities in the mosquito midgut has been shown to affect both *Plasmodium* development and mosquito immunological signaling (Zheng et al., 2023; Liu et al., 2024). Gut bacteria, such as *Staphylococcus aureus, Staphylococcus epidermidis, Wolbachia* spp.*, Serratia* spp., and *Asaia* spp., have been found to produce bioactive compounds and activate immune pathways, which inhibit the development of the parasite and the physiological conditions that promote *Plasmodium* sporogony (Romoli and Gendrin, 2018; Gao et al., 2021; Zheng et al., 2023). These complex three-way interactions between the mosquito host, its microbiota, and invasive Plasmodium parasites have been radically reconceptualized by the use of metagenomic tools, such as 16S rRNA amplicon sequencing and shotgun metagenomics (Sharpton, 2014; Ortiz‐Estrada et al., 2019).

These are just some of the developments that have occurred to this point, yet there are still key gaps in knowledge. Current studies of this kind have tended to be descriptive, listing individual microbial taxa and mechanisms without presenting a systematic overview of the extent, depth and quality of the available evidence base. The spectrum of metagenomic methods used, the distribution of evidence across mosquito species and geographic location, and the diversity of microbiota functions have not yet been fully characterized by a scoping review. Any such synthesis is critical to uncovering inconsistencies in the literature, outlining poorly explored aspects of the body of work, and to prioritize future studies and translation. This scoping review, therefore, seeks to fill this gap by systematically charting current scientific studies on the contribution of the mosquito gut microbiome to the development of Plasmodium parasites, and the impacts of metagenomic approaches on vector-based malaria control. The review is guided by the following research question, framed using the Population-Concept-Context (PCC) framework as recommended for scoping reviews:

Population: Mosquito species relevant to *Plasmodium* transmission

Concept: The composition, acquisition, function, and manipulation of the mosquito gut microbiome

Context: *Plasmodium* parasite growth, vector competence, and microbiome-based malaria prevention techniques, such as metagenomic surveillance and paratransgenesis

The core research question is: What is the scope, nature, and extent of evidence on the mosquito gut microbiome’s role in *Plasmodium* parasite development, as assessed through metagenomic approaches, and how can this evidence inform sustainable malaria vector control?

### Approaches used to characterize the mosquito gut microbiome

#### 16S rRNA metagenomics

The 16S rRNA sequencing method is a widely utilized and effective technique for analyzing the gut microbiome of mosquitoes. The 16S ribosomal RNA gene, which has nine hypervariable regions (V1–V9) that offer species-level identification and conserved areas helpful for primer design, is amplified using this technique (Ortiz‐Estrada et al., 2019). Researchers can now identify uncommon microbial taxa even in complicated habitats like the mosquito gut, thanks to the use of Next-Generation Sequencing technologies, which have improved the sensitivity and cost-effectiveness of sequencing 16S rRNA (Ortiz‐Estrada et al., 2019). The process includes sample collection, DNA extraction, PCR amplification with forward and reverse primers, library preparation, and sequencing on platforms such as Oxford Nanopore or Illumina, as shown in **Figure 1**. Following sequencing, bacterial taxonomy at the species, genus, and family levels is determined by clustering sequences into operational taxonomic units (OTUs) and comparing them to reference databases (Johnson et al., 2019). Both sensitivity and resolution have been enhanced by next-generation sequencing. Long-read technologies, such as Oxford Nanopore, can sequence the full length of the 16S gene (V1-V9) for species-level resolution; short-read technologies, such as Illumina, which sequences a partial region (V3-V4) for genus-level classification (Aja-Macaya et al., 2025). This has shown to be a promising method for detecting significant taxonomic diversity in mosquito gut microbiota, which is crucial for microbiomics studies in vector biology (Díaz et al., 2021).

**Figure 1:**
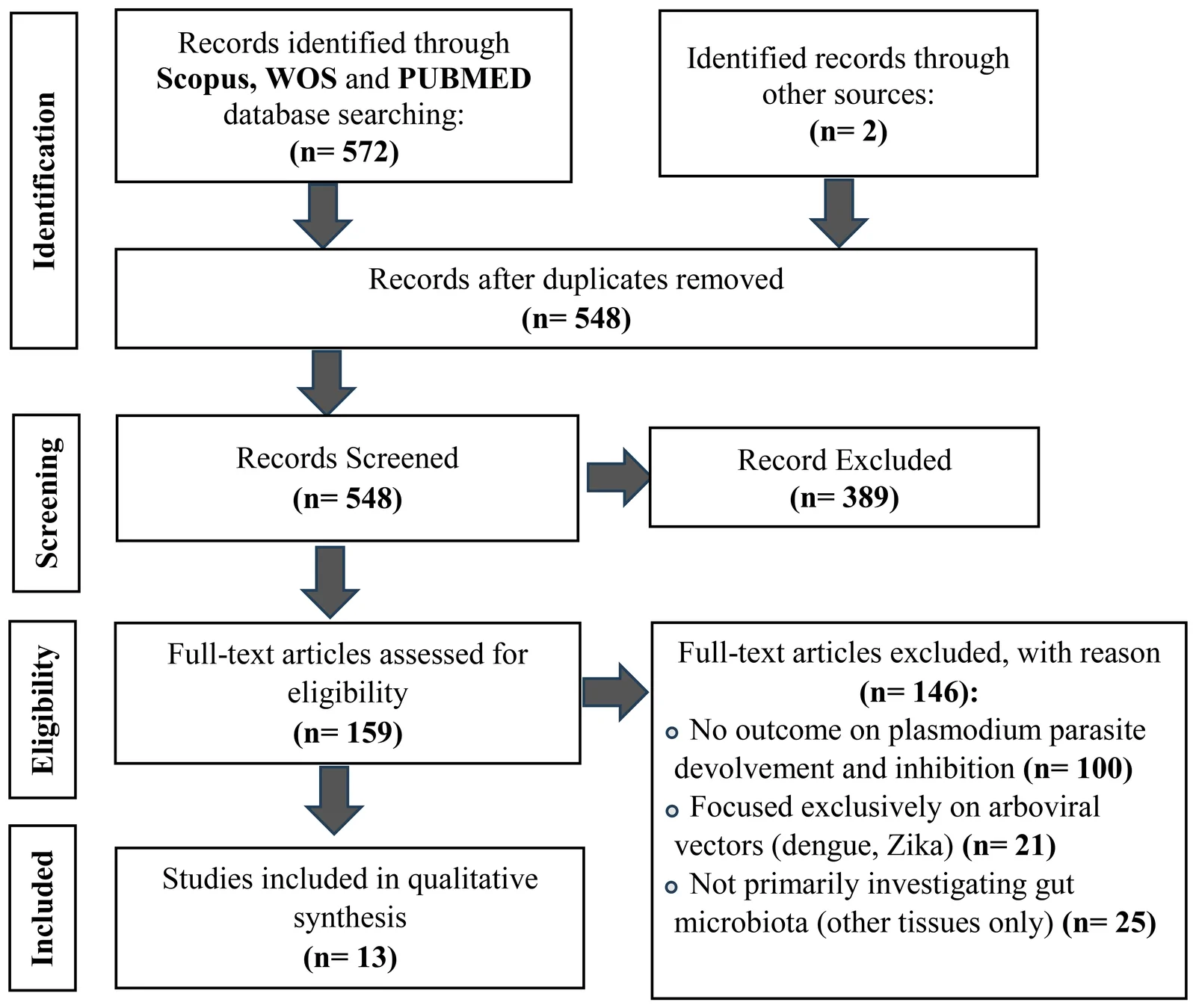
Flowchart of the article search and screening process.

#### Shotgun metagenomics sequencing

Targeted approaches to the study of uncultured microbiota face limitations, and shotgun metagenomic sequencing (SMS) can tackle them. It involves the removal of DNA from a community of microbes, disruption, and sequencing of individual DNA segments (Sharpton, 2014). This yields multiple types of DNA reads, some representing protein-coding regions providing insights into functionality, and others with a functional association to taxonomically relevant regions such as the 16S rRNA gene (Sharpton, 2014). This method offers a dual benefit by determining the microbial makeup and their biological roles (Sharpton, 2014). By capturing genetic data from various organisms, including non-microbial species, shotgun metagenomics enables researchers to analyze both the diversity and functional potential of a microbial ecosystem, be it viruses, bacteria, and fungi (Sharpton, 2014).

The first step in a mosquito population’s metagenomic investigation is to extract DNA from each sample’s cells. After that, a highly conserved genomic marker is selected and amplified using PCR to help with identification (Tiwari et al., 2022). The next step is binning, which involves classifying sequences into taxonomic groups by either clustering them according to shared traits or comparing them to reference databases (Tiwari et al., 2022). This process simplifies data complexity, evaluates microbial diversity, and finds new genomes. Lastly, rather than using fragmented short reads, assembly creates larger segments that improve bioinformatic analysis by combining overlapping reads from the same genome into consensus sequences (Tiwari et al., 2022).

## Methodology

### Study design and framework

This study was designed as a scoping review, a methodological approach appropriate for mapping the extent, range, and nature of evidence on a given topic, identifying key concepts and knowledge gaps, and reporting on evidence regardless of study quality (Arksey and O’Malley, 2005; Tricco et al., 2018). The review was conducted in accordance with the Arksey and O’Malley (2005), five-stage scoping review framework, as refined by Tricco (Tricco et al., 2018), and reported following the PRISMA Extension for Scoping Reviews (PRISMA-ScR) checklist.

### Eligibility criteria

**Inclusion criteria:** Studies were eligible for inclusion if they: (1) investigated the gut microbiome or specific gut microbial taxa of mosquito species relevant to *Plasmodium* transmission; (2) assessed the relationship between gut microbiota and *Plasmodium* parasite development, vector competence, or mosquito immune responses; (3) employed microbiological, molecular, or metagenomic methods including 16S rRNA sequencing, shotgun metagenomics, culture-based isolation, or paratransgenic approaches; (4) were published as peer-reviewed primary research articles, systematic reviews, or comprehensive narrative reviews; (5) were published in the English language.

**Exclusion criteria:** Studies were excluded based on the following criteria: (I) studies focusing exclusively on mosquito-borne arboviruses with no primary investigation of *Plasmodium* biology or transmission; (II) studies examining arthropod vectors other than mosquitoes; (III) reported purely computational or modelling studies without biological validation of microbiome-parasite interactions; (IV) were conference abstracts, commentaries, editorials, or letters without substantive data; (V) articles for which full-text versions could not be retrieved after two independent attempts using multiple databases.

### Search strategy and databases

A systematic literature search was conducted in the following electronic databases: PubMed https://pubmed.ncbi.nlm.nih.gov/), Scopus (https://www.scopus.com/), and Web of Science (https://www.webofscience.com/). Additional searches were performed in grey literature sources, and the reference lists of eligible articles were manually screened for further relevant studies. The search was conducted in English, with no language exclusions applied during initial retrieval. All searches were performed by two independent reviewers to minimize selection bias.

The following representative Boolean search strings were applied across databases: (“mosquito gut microbiome” OR “mosquito microbiota”) AND (*“Plasmodium”* OR “malaria parasite”) AND (“vector competence” OR “parasite development”) AND (“Anopheles” OR “Culex”) AND (“gut bacteria” OR “gut microbiome” OR “microbiota”) AND (*“Plasmodium falciparum”* OR *“Plasmodium berghei”*) AND (“paratransgenesis” OR “symbiotic bacteria”) AND (“16S rRNA” OR “shotgun metagenomics” OR “metagenomic sequencing”). The search encompassed publications from 01/01/2000 to 30/04/2026.

### Study selection process

Following database searching and de-duplication of retrieved records using Rayyan online server (https://www.rayyan.ai/), two independent reviewers screened all records at the title and abstract level against the inclusion and exclusion criteria. Full texts of potentially eligible records were retrieved and assessed independently by the same reviewers. Discrepancies between reviewers at both stages were resolved through discussion and, where necessary, arbitration by a third reviewer. The study selection process was described in accordance with the PRISMA-ScR flow diagram and summarized in **Figure 1**. Reasons for exclusion at the full-text stage were documented.

### Data and extraction method

Data were extracted by one reviewer and verified by a second. The following variables were extracted from each eligible study: Study identification details (author(s), year, country); Mosquito species; Methodological approach (16S rRNA, shotgun metagenomics, culture-based, paratransgenic); Bacteria species identified; *Plasmodium* species tested; Experimental method; Outcome.

### Data synthesis

Given the heterogeneity of study designs, methods, and outcomes across the included literature, a quantitative meta-analysis was not appropriate. Data synthesis was conducted through thematic analysis and descriptive mapping, consistent with the scoping review methodology. Identified studies were categorized into thematic domains based on the primary focus of each study, and evidence was mapped according to mosquito species, *Plasmodium* species, geographic region, methodological approach, and direction of microbial effect on parasite development. Where appropriate, findings were presented in structured summary tables to facilitate comparative analysis across studies.

## Results

### Study selection and PRISMA-ScR flow

When the database was initially searched on PubMed, Scopus and Web of Science, 572 records were identified, with another 2 records found by scanning the reference lists, bringing the total number of records to 574. Based on the Rayyan platform, 548 different abstracts and titles were filtered. At the title/abstract stage, 389 of these records were eliminated because they did not fit the eligibility criteria. For the remaining 159 records, full texts were obtained and evaluated. Thirteen primary studies were included in the final qualitative synthesis after 146 articles were eliminated after full-text review. The complete selection process is summarized in the PRISMA-ScR flow diagram (**Figure 1**). This scoping review synthesized evidence from thirteen studies varying in country of origin, mosquito species, sequencing methodology, body region sampled, bacterial taxa identified, and Plasmodium species tested, as summarized in **Table 1**.

**Table 1:** Summary of data from the thirteen eligible studies.

Author(s) & Year	Country	Mosquito specie	Sequencing method	Mosquito body part/region analyzed	Bacterial Species identified	*Plasmodium* species tested	Experimental method	Outcome
Bassene et al. (2018)	Senegal	*Anopheles gambiae s.l.* *Anopheles funestus*	Illumina MiSeq (16S rRNA V3-V4, paired-end 2×250 bp)	Abdomen	*Burkholderia* spp. (49.96%, dominant overall; *B. fungorum*, *B. cepacia* in Pf-free samples), *Sphingomonas* (14.5%), *Bacillus* (11.20%), *Pseudomonas* (4.24%), *Asaia* aff. *bogorensis*, *Enterobacter cloacae*, *Elizabethkingia meningoseptica*, *Wolbachia* sp., *Sodalis* sp., *Bartonella* spp.; Dominant phyla: Proteobacteria (53%), Firmicutes (31.34%)	*Plasmodium falciparum* (Pf)	Observational 16S metagenomic comparison of naturally Pf-infected vs. Pf-uninfected wild-caught mosquitoes; correlation and PCA analysis of bacterial genera vs. infection status	Pf-infected mosquitoes exhibited greater bacterial diversity than non-infected mosquitoes. *Burkholderia, Sphingomonas, Bosea, Bradyrhizobium*, and *Asaia* were negatively associated with Pf infection, whereas *Pantoea, Aeribacillus, Microvirga*, and *Micrococcus* were positively associated with Pf carriage, indicating potential microbiome-mediated effects on parasite establishment
Sharma et al. (2020)	India	*Anopheles stephensi*	Illumina MiSeq (16S rRNA V3-V4) + RNAseq (Illumina NextSeq)	Midgut	*Elizabethkingia meningoseptica* (dominant), *Pseudomonas* spp., *Serratia* spp.; also, *Wolbachia* endosymbiont detected; dominant phyla: Bacteroidetes (73.13%), Proteobacteria (16.4%)	*Plasmodium vivax*	Observational + infection assay: Metagenomics and RNAseq of naïve, blood-fed, and *P. vivax*-infected mosquito; time-course monitoring of bacterial abundance via RT-qPCR post-infection; antimicrobial peptide (AMP) expression profiling	*P. vivax* infection suppressed the total *A. stephensi* gut microbiota, including the dominant *Elizabethkingia* and *Serratia*, and gut antimicrobial peptides (cecropin-1/-2, defensin-1, gambicin) below detection for ~36 h, with both rebounding thereafter (p < 0.005), suggesting the parasite dampens microbiota-mediated immunity during the pre-invasive window to favor its own survival
Tchioffo et al. (2016)	Cameroon	*Anopheles gambiae*/*An. coluzzii*	454 pyrosequencing (16S rRNA V3-V4)	Midgut, ovaries, salivary glands	*Pseudomonas*, *Comamonas*, *Acinetobacter*, *Rhizobium*, *Burkholderia*, *Serratia*, *Asaia*, *Elizabethkingia*, Enterobacteriaceae	*Plasmodium falciparum*	Observational; mosquitoes dissected at emergence, 24 h, and 8 days post-infectious blood meal; bacterial community compared between infected and uninfected mosquitoes	*P. falciparum-infected An. gambiae/coluzzii* showed significantly higher midgut and salivary-gland abundance of *Serratia*, and higher midgut abundance of *Methylobacterium*, than uninfected mosquitoes given the same infectious blood meal, indicating both taxa are positively associated with infection status without evidence of a causal role.
Kalappa et al. (2018)	India	*An. stephensi*	Illumina MiSeq (16S rRNA V3 region)	Midgut	*Acinetobacter*, *Pseudomonas*, *Prevotella*, *Corynebacterium*, *Veillonella*, *Bacillus*; Dominant phylum: Proteobacteria	*Plasmodium berghei*	Infection assay: antibiotic-treated vs. untreated mosquitoes fed on *P. berghei*-infected mice; oocyst counts on day 10	Experimental clearance of the native midgut microbiota by antibiotic treatment significantly increased both the intensity (oocyst load) and prevalence of *P. berghei* infection across all three geographically distinct *An. stephensi* strains, relative to untreated mosquitoes retaining their natural microbiota, providing direct experimental evidence that the total gut microbiota confers a protective, parasite-inhibitory effect on *Plasmodium* development. The microbiota was dominated by *Proteobacteria* (genera *Acinetobacter*, *Pseudomonas*, *Prevotella*, *Corynebacterium*, *Veillonella*, and *Bacillus*), but the inhibitory effect was attributed to the community as a whole, with no individual taxon linked to the parasite outcome
Surat et al. (2016)	Thailand	*An. minimus*	454 pyrosequencing (16S rRNA V3-V4)	Gut	*Acinetobacter*, *Burkholderia*, *Thorsellia*, *Serratia*, *Elizabethkingia*, *Pseudomonas*, *Klebsiella*, *Enterobacter*, *Delftia*; Dominant phyla: Proteobacteria (Gamma- and Betaproteobacteria	*Plasmodium falciparum*	Culture-independent comparison of gut microbiota between naturally *Plasmodium*-infected and uninfected field-caught mosquitoes	The gut microbiota of *P. falciparum*-infected and uninfected *An. minimus* differed in diversity and composition: uninfected mosquitoes had higher bacterial richness and were dominated by betaproteobacteria (*Burkholderia*, *Alcaligenes*, and *Serratia*), which were therefore potentially inhibitory, whereas infected mosquitoes were dominated by gammaproteobacteria (*Acinetobacter*, *Thorsellia*, and unclassified Enterobacteriaceae), which were potentially promotive. These directions reflect observational associations only, with no causal effect on parasite development demonstrated
Sandeu et al. (2025)	Cameroon	*Anopheles gambiae*	Illumina MiSeq (16S rRNA V3-V4, DADA2/QIIME2)	Whole mosquito	*Klebsiella, Asaia, Pseudomonas, Serratia, Acinetobacter, Enterobacter, Pantoea; Proteobacteria dominant (64.8%)*	*Plasmodium falciparum*	Observational; microbiota composition compared between naturally infected and uninfected mosquitoes across wet and dry seasons using LEfSe and PERMANOVA	Microbiota composition differed significantly between *P. falciparum*-infected and uninfected *An. gambiae*, with 14 bacterial genera more abundant in uninfected mosquitoes (potentially inhibitory) and only 2 more abundant in infected mosquitoes (potentially promotive); *Acetobacteraceae* and *Enterococcus* were unique to uninfected mosquitoes, while none were exclusive to infected ones.
Boissière et al. (2012)	Cameroon	*Anopheles gambiae (M and S molecular forms)*	454 pyrosequencing (16S rRNA, 3 hypervariable regions)	Midgut	*Asaia, Burkholderia, Serratia, Ralstonia, Acinetobacter, Pseudomonas, Sphingomonas, Enterobacteriaceae (unclassified), Elizabethkingia (laboratory colony)*	*Plasmodium falciparum*	Observational; Midgut microbiota of field-collected mosquitoes experimentally challenged with *P. falciparum*; infected vs uninfected midguts compared by RDA and Mann–Whitney test	The relative abundance of *Enterobacteriaceae* in the midgut was significantly higher in *P. falciparum*-infected than uninfected *An. gambiae* (Mann-Whitney P = 0.004), identifying *Enterobacteriaceae* as positively associated with infection status (potentially promotive)
Tchioffo et al. (2013)	Cameroon	*Anopheles gambiae*	Sanger sequencing (16S rRNA; culture-dependent MacConkey agar)	Midgut	*Proteobacteria only: Escherichia/Shigella, Serratia marcescens, Pseudomonas stutzeri, Enterobacter* spp.*, Acinetobacter septicus, Comamonas* spp.*, Aeromonas hydrophila, Klebsiella* spp.*, Delftia* spp.*, Rahnella aquatilis; Enterobacteriaceae dominant (78%)*	*Plasmodium falciparum*	Infection assay; laboratory mosquitoes orally challenged with isolated bacterial strains (10^9^ CFU/ml via sugar meal, 36 h pre-infection), then membrane-fed on natural *P. falciparum* gametocyte carriers; oocysts counted at 8 days post-blood meal; GLMMs used for analysis	Experimental oral challenge with natural midgut bacteria isolated from field *An. gambiae* significantly reduced both the prevalence and intensity of *P. falciparum* infection, providing direct experimental evidence that specific gut bacteria, most notably *Escherichia coli*, *Serratia marcescens*, and *Pseudomonas stutzeri*, inhibit parasite development in the mosquito and could be exploited as candidates for microbiota-based malaria transmission control
(Gao et al., 2021)	China	*Anopheles gambiae*	16S rDNA amplicon sequencing (V3-V4 region, Illumina MiSeq); plus culture-based 16S rDNA identification of isolates and whole-genome sequencing	Midgut	*Asaia* and *Serratia* in *Yunnan, Serratia and Rhodococcus* in *Liaoning* (LN1), *Arcobacter* and *Serratia in Jiangsu* (JS1). Ten genera were shared across all three populations: *Serratia, Pseudomonas, Acinetobacter, Cupriavidus, Propionibacterium, Corynebacterium, Methylobacterium, Flavobacterium, Lactobacillus*, and *Oscillospira*, with *Serratia* the most abundant shared symbiont	*P. falciparum and P. berghei, P. vivax* and *P. yoelii*	Gut symbionts isolated from field *An. sinensis* and characterized by 16S profiling and whole-genome sequencing; selected strains fed to *An. stephensi* and *An. gambiae* before *Plasmodium* challenge, with oocyst prevalence and intensity compared against controls; antimalarial factor identified by mass spectrometry, gene knockout, complementation, and purified-protein killing assays	Gut symbiont *Serratia ureilytica* Su_YN1 secretes an antimalarial lipase (AmLip) that directly kills parasites and inhibits *P. falciparum* and *P. berghei* oocyst formation by up to 98-100%; active-site mutation abolished and complementation restored the effect, establishing causation. A second strain (*S. marcescens* Sm_YN3) inhibits parasites indirectly via Toll immune activation, while other *Serratia* strains had no effect, demonstrating strain-specific interactions. Su_YN1 self-disseminates through mosquito populations by vertical, trans-stadial, and venereal transmission
(Feng et al., 2021)	China	*An. stephensi*	16S rRNA pyrosequencing (V3-V4, MiSeq) for community profiling; whole-genome sequencing (Illumina NovaSeq); plus RNA-seq	Midgut	Dominant genera: *Enterobacter, Pseudomonas, Gluconobacter, Acinetobacter, Vibrio, Tatumella, Photobacterium, Ralstonia, Sphingomonas.* Isolated/characterized: *Pseudomonas alcaligenes*	*P. berghei (ANKA)*	Experimental: antibiotic microbiota depletion, LC-MS metabolomics, metabolite supplementation, RNAi (HKT), 16S profiling, whole-genome sequencing and KynU knockout of Pseudomonas alcaligenes with re-colonization, measuring *P. berghei* oocyst load and peritrophic matrix integrity in *An. stephensi*.	Gut microbiota of *Anopheles stephensi* participates in mosquito tryptophan metabolism, and its removal caused the tryptophan metabolite 3-hydroxykynurenine (3-HK) to accumulate, impairing the peritrophic matrix and increasing *P. berghei* oocyst development. The commensal bacterium *Pseudomonas alcaligenes* prevents this by degrading 3-HK through its kynureninase (KynU) enzyme; deleting KynU abolished 3-HK degradation and largely removed the protective effect, demonstrating that *P. alcaligenes* inhibits parasite development indirectly by preserving midgut barrier integrity
(Carlos et al., 2025)	Peru	*Anopheles darlingi*	Host midgut RNA-seq (Illumina NextSeq 500) + 16S rRNA V4 amplicon sequencing (Illumina MiSeq); 16S qRT-PCR for bacterial load	Midgut	Enriched in low-infection mosquitoes: *Pantoea*, *Serratia*, *Thorsellia*, *Pseudomonas*, *Acinetobacter*. Enriched in high-infection mosquitoes: *Elizabethkingia*, *Chryseobacterium*.	*P. vivax*	Observational; colony *An. darlingi* infected with field *P. vivax* by membrane feeding (heat-inactivated controls), midguts sampled across six timepoints; paired host midgut RNA-seq and 16S rRNA V4 microbiota profiling comparing high versus low oocyst-burden phenotypes	In *P. vivax*-infected *An. darlingi*, mosquitoes with low oocyst burden were enriched for *Pantoea*, *Serratia*, *Thorsellia*, *Pseudomonas*, and *Acinetobacter* (potentially inhibitory) and showed reduced bacterial diversity with a metabolically restrictive midgut, whereas high-burden mosquitoes were enriched for *Elizabethkingia* and *Chryseobacterium* (potentially promotive). Associations only; the authors caution against causal inference given the small sample size
(Bahia et al., 2014)	USA	*An. gambiae*		Midgut	*Serratia marcescens*, *Elizabethkingia anophelis*, *Pseudomonas putida*, *Pseudomonas rhodesiae*, *Pantoea* sp., *Acinetobacter* sp., and *Comamonas* sp.	*P. falciparum* and *P. berghei*	Experimental; seven 16S-identified gut bacterial isolates introduced into *An. gambiae* by blood meal, sugar meal, or injection; midgut colonization tracked, anti-*Plasmodium* activity tested *in vivo* and *in vitro*, IMD pathway tested by RNAi, and mosquito survival assessed	Seven *Anopheles* gut bacterial isolates experimentally inhibited *P. falciparum* development, both *in vivo* (reduced oocyst load) and *in vitro*. *Serratia marcescens* was most effective, blocking both sexual and asexual stages via a secreted factor and also shortening mosquito lifespan, with *Pseudomonas putida* and *Pantoea* sp. also significantly reducing infection. Inhibition acted through several mechanisms (direct interaction, secreted factors, and IMD immune activation), marking these bacteria, especially *S. marcescens*, as candidates for microbiota-based transmission-blocking control
(Gendrin et al., 2015)	UK	*Anopheles gambiae*	16S rDNA 454-pyrosequencing (regions V2-V3) for community composition, plus 16S rDNA qPCR (generic and taxon-specific) for bacterial load	Midgut	*Community dominated by Flavobacteriaceae (Chryseobacterium, Elizabethkingia) and Enterobacteriaceae (Cedecea, Enterobacter, Ewingella, Serratia); also Aeromonas, Comamonas, Burkholderia, and Asaia (Acetobacteraceae)*	*P. berghei* and *P. falciparum*	*An. gambiae* were fed blood supplemented with therapeutic penicillin/streptomycin (PS) or control, across up to three blood feeds via membrane feeder; midgut microbiota load and composition were tracked by 16S qPCR and 454-pyrosequencing over time; mosquito susceptibility to *P. berghei* and *P. falciparum* (lab and field) was measured by oocyst prevalence and intensity; and effects on mosquito survival and fecundity were assessed (GLMM statistics)	Antibiotics (penicillin/streptomycin) present in ingested blood disrupted the *An. gambiae* gut microbiota, halving bacterial diversity and sharply reducing Enterobacteriaceae (notably *Serratia*) while allowing *Asaia* to expand, and this significantly increased mosquito susceptibility to *Plasmodium*, raising both prevalence and intensity of *P. berghei* and *P. falciparum* infection (confirmed with naturally infected field blood). Antibiotic exposure also increased mosquito survival and fecundity

### Thematic synthesis of findings

Synthesis of the 13 included studies identified four themes. The first two organize the bacterial taxa by the direction of their relationship with *Plasmodium*, separating taxa that inhibit or are inversely associated with parasite development from those that are positively associated with infection. The third addresses the divergent patterns of microbial diversity reported during infection, and the fourth maps the methodological landscape and the resulting evidence gaps. Throughout, experimental evidence is distinguished from observational association, and associative wording is retained wherever causation was not demonstrated.

### Gut microbiota negatively associated with *Plasmodium* development

The largest and most consistent body of evidence concerns bacteria that inhibit, or are inversely associated with, parasite development. This evidence separates into experimental demonstrations of inhibition and observational associations with the uninfected or lower-burden state.

### Experimental evidence

Two studies demonstrated protection at the level of the whole community. Kalappa (Kalappa et al., 2018) showed that antibiotic clearance of the native midgut microbiota significantly increased both the prevalence and intensity of *P. berghei* infection across three geographically distinct *An. stephensi* strains, indicating that the intact community as a whole is protective. Gendrin (Gendrin et al., 2015) reached a concordant conclusion from the opposite direction, demonstrating that penicillin and streptomycin ingested in a blood meal disrupted the *An. gambiae* microbiota, halving bacterial diversity, and sharply reducing the Enterobacteriaceae, notably *Serratia*, while increasing susceptibility to both *P. berghei* and *P. falciparum*.

Four further studies demonstrated inhibition attributable to specific taxa. Tchioffo (Tchioffo et al., 2013) fed cultured midgut isolates to mosquitoes before an infectious blood meal and showed that *Escherichia coli*, *Serratia marcescens*, and *Pseudomonas stutzeri* significantly reduced *P. falciparum* prevalence and oocyst intensity, whereas *Acinetobacter septicus* had no effect on prevalence. Bahia (Bahia et al., 2014) found that seven gut isolates reduced *P. falciparum* development, with *Serratia marcescens* the most effective, acting on both sexual and asexual stages through a secreted factor, and with *Pseudomonas putida* and *Pantoea* sp. also significantly reducing infection. Gao (Gao et al., 2021) identified the mechanism at molecular resolution, showing that *Serratia ureilytica* strain Su_YN1 secretes an antimalarial lipase that directly kills parasites and inhibits *P. falciparum* and *P. berghei* oocyst formation by 98-100%, with an active-site mutation abolishing the effect and complementation restoring it. Feng (Feng et al., 2021) demonstrated an indirect metabolic route in which the commensal *Pseudomonas alcaligenes* degrades the tryptophan metabolite 3-hydroxykynurenine via its kynureninase enzyme, thereby preserving peritrophic matrix integrity; loss of the bacterium or its kynureninase gene increased *P. berghei* infection.

### Observational evidence

Four observational studies reported taxa enriched in uninfected or lower-burden mosquitoes. Bassene (Bassene et al., 2018) found *Burkholderia*, *Sphingomonas*, *Bosea*, *Bradyrhizobium*, and *Asaia* negatively associated with *P. falciparum* carriage. Surat (Surat et al., 2016) reported that uninfected *An. minimus* is dominated by the betaproteobacteria *Burkholderia*, *Alcaligenes*, and *Serratia*. Sandeu (Sandeu et al., 2025) found 14 genera more abundant in uninfected *An. gambiae*, with *Acetobacteraceae* and *Enterococcus* detected only in uninfected individuals. Carlos (Carlos et al., 2025) observed that *An. darlingi* with a low *P. vivax* oocyst burden were enriched for *Pantoea*, *Serratia*, *Thorsellia*, *Pseudomonas*, and *Acinetobacter*.

### Reported mechanisms

The experimental studies collectively describe several distinct mechanisms of protection: direct antiparasitic activity through secreted factors, most clearly the lipase, characterized by (Gao et al., 2021) and the secreted activity of *Serratia marcescens* reported by (Bahia et al., 2014) and (Tchioffo et al., 2013); activation of mosquito immune signaling, with (Bahia et al., 2014) attributing part of the effect of some isolates to the IMD pathway; community-level protection revealed by microbiota depletion in (Kalappa et al., 2018) and (Gendrin et al., 2015); and preservation of midgut barrier integrity through microbial regulation of host metabolism in (Gao et al., 2020).

### Gut microbiota positively associated with *Plasmodium* infection

Several studies identified taxa enriched in infected mosquitoes or correlated with higher parasite burden. All such findings were observational, and none demonstrated that these taxa promote infection; the evidence therefore supports association rather than causation. Boissiere (Boissière et al., 2012) found the *Enterobacteriaceae* significantly more abundant in *P. falciparum*-infected midguts. Tchioffo (Tchioffo et al., 2016) reported *Serratia* and *Methylobacterium* enriched in infected midguts and salivary glands at day 8 after an infectious blood meal. Bassene (Bassene et al., 2018) found *Pantoea*, *Aeribacillus*, *Microvirga*, and *Micrococcus* positively associated with *P. falciparum* carriage. Surat (Surat et al., 2016) observed that infected *An. minimus* guts were dominated by the gammaproteobacteria *Acinetobacter*, *Thorsellia*, and unclassified *Enterobacteriaceae*. Sandeu (Sandeu et al., 2025) reported two genera that were more abundant in infected than uninfected *An. gambiae*. Carlos (Carlos et al., 2025) found *Elizabethkingia* and *Chryseobacterium* enriched in *An. darlingi* with a high oocyst burden.

### Microbial diversity patterns during infection

The included studies reported divergent relationships between microbial diversity and infection, and the direction was not consistent. Bassene (Bassene et al., 2018) reported higher bacterial diversity in *P. falciparum*-infected *An. gambiae* and *An. funestus*. In contrast (Surat et al., 2016), found 1.30 to 1.64 times higher richness in uninfected *An. minimus*, and (Sandeu et al., 2025) reported compositional differences between infected and uninfected *An. gambiae* whose direction varied between wet and dry seasons. Carlos (Carlos et al., 2025) found that low-burden *An. darlingi* carried reduced diversity, a direction opposite to that reported by (Surat et al., 2016). Sharma (Sharma et al., 2020) described a temporal pattern rather than a static difference, observing that total gut bacterial abundance, including the dominant *Elizabethkingia* and *Serratia*, declined below detection for approximately 36 hours after an infectious *P. vivax* blood meal and rebounded thereafter. Two studies indicated that factors other than infection shaped community structure, with (Boissière et al., 2012) and (Bassene et al., 2018) both finding larval breeding-site ecology to be the primary determinant of composition.

These conflicting patterns are most plausibly explained by differences among studies rather than by a single underlying relationship: the mosquito species examined (*An. gambiae*, *An. funestus*, *An. stephensi*, *An. minimus*, *An. darlingi*), the parasite species (*P. falciparum*, *P. vivax*, *P. berghei*), the contrast measured (infection status versus oocyst burden versus a post-infection time course), the tissue sampled (dissected midgut versus whole mosquito, as in (Sandeu et al., 2025), the sequencing platform (454 pyrosequencing versus Illumina), and, in (Sandeu et al., 2025), the season of collection.

### Current methodological landscape and evidence gaps

The evidence base rested predominantly on 16S rRNA amplicon sequencing, which was used in the large majority of the 13 studies to profile community composition. Older 454 pyrosequencing was used by four studies (Tchioffo et al., 2016; Gendrin et al., 2015; Bahia et al., 2014; Surat et al., 2016), and current Illumina platforms by the remainder, and only (Sandeu et al., 2025) applied an amplicon sequence variant pipeline (DADA2 or QIIME2) rather than operational taxonomic unit clustering. Two studies relied on culture-dependent isolation with Sanger identification (Bahia et al., 2014; Tchioffo et al., 2013). A minority extended beyond amplicon profiling: whole-genome sequencing of isolates was used by Feng et al. (2021), host or microbial transcriptomics by Sharma et al., 2020, Feng et al. (2021), and Carlos et al. (2025), and metabolomics by Feng et al. (2021). Notably, no study applied shotgun metagenomic profiling of the intact community. By study design, six studies were experimental (Kalappa et al., 2018; Gendrin et al., 2015; Bahia et al., 2014; Tchioffo et al., 2013; Feng et al., 2021; Gao et al., 2021) and seven were observational (Tchioffo et al., 2016; Carlos et al., 2025; Sandeu et al., 2025; Bahia et al., 2014; Sharma et al., 2020; Surat et al., 2016; Bassene et al., 2018), so that direct functional or mechanistic evidence was confined to a minority of the corpus.

In summary, the synthesis indicates that the mosquito gut microbiota is, on balance, inhibitory to *Plasmodium* development, an inference supported directly by experimental depletion and challenge studies and consistent with the observational enrichment of specific taxa in uninfected mosquitoes. A smaller set of taxa is positively associated with infection, but only on observational evidence. Reports of microbial diversity in relation to infection are contradictory and appear to be governed by study-specific ecological and methodological factors. The near-exclusive reliance on 16S rRNA amplicon sequencing and the absence of shotgun metagenomics constrain the field to taxonomic association and leave the functional basis of most reported relationships undetermined.

## Discussion

### Overview of principal findings

This review mapped 13 primary studies of the mosquito gut microbiome and *Plasmodium* development. Four findings dominate. First, the microbiota is, on balance, inhibitory to parasite development: experimental depletion increased infection (Kalappa et al., 2018; Gendrin et al., 2015), and challenge with specific isolates reduced it (Bahia et al., 2014; Tchioffo et al., 2013; Gao et al., 2021). Second, the taxa most consistently associated with reduced infection, either experimentally or observationally, include *Serratia*, *Pseudomonas*, *Burkholderia*, and *Asaia*, together with the community as a whole. Third, a distinct set of taxa, including the *Enterobacteriaceae*, *Elizabethkingia*, *Chryseobacterium*, *Methylobacterium*, *Pantoea*, and several others, is positively associated with infection, but only in observational studies. Fourth, the field rests predominantly on 16S rRNA amplicon sequencing, with a minority of studies adding whole-genome sequencing, transcriptomics, or metabolomics, and none applying shotgun metagenomics.

The strongest evidence in this review is experimental. Gendrin and Kalappa (Kalappa et al., 2018; Gendrin et al., 2015) independently showed that removing or disrupting the microbiota increases *Plasmodium* infection, establishing a protective role for the intact community and, importantly, attributing this effect to the community rather than to any single taxon. Bacterial-challenge studies then localized inhibition to specific organisms: Tchioffo and Bahia (Bahia et al., 2014; Tchioffo et al., 2013) both identified *Serratia marcescens* as strongly inhibitory, and Gao (Gao et al., 2021) defined a mechanism, a secreted antimalarial lipase, at the level of a single strain. Feng (Feng et al., 2021) added a mechanistically distinct route in which a commensal *Pseudomonas* protects the peritrophic matrix by metabolizing a host-derived toxin. The areas of agreement are substantial: multiple independent studies, across *An. gambiae*, *An. stephensi*, and *An. sinensis*, converge on a protective microbiota and on *Serratia* and *Pseudomonas* as inhibitory candidates.

The observational studies are broadly concordant but yield weaker inferences (Carlos et al., 2025; Sandeu et al., 2025; Surat et al., 2016; Bassene et al., 2018), each reported taxa enriched in uninfected or low-burden mosquitoes, and several of these taxa (*Burkholderia*, *Pseudomonas*, *Serratia*, *Asaia*) coincide with the experimentally inhibitory set, strengthening the overall inference. The principal contradiction lies within the *Serratia* and *Enterobacteriaceae* group, which is inhibitory in experimental studies (Bahia et al., 2014; Tchioffo et al., 2013; Gao et al., 2021), yet positively associated with infection in observational studies (Tchioffo et al., 2016; Bahia et al., 2014). Gao (Gao et al., 2021) offer the most coherent reconciliation, demonstrating that different *Serratia* species, and even different strains of one species, exert opposite effects depending on whether they carry a functional antimalarial gene. This strain-level heterogeneity, invisible to 16S rRNA sequencing, is the most parsimonious explanation for the apparent contradiction.

A recurring set of taxa was enriched in infected mosquitoes or in those with higher parasite burden: the *Enterobacteriaceae* (Boissière et al., 2012), *Serratia* and *Methylobacterium* (Tchioffo et al., 2016), *Pantoea*, *Aeribacillus*, *Microvirga*, and *Micrococcus* (Bassene et al., 2018), *Acinetobacter* and *Thorsellia* (Surat et al., 2016), and *Elizabethkingia* and *Chryseobacterium* (Carlos et al., 2025). It is essential to state that all of these are observational associations. No included study experimentally demonstrated that any of these taxa promotes infection, and the wording used here, enriched in infected mosquitoes or associated with higher burden, reflects that limitation. The strength of the evidence is modest: sample sizes were often small, the contrasts were cross-sectional, and infection status may itself reshape the microbiota, so that enrichment in infected mosquitoes may be a consequence rather than a cause of infection. The bidirectional behavior of *Serratia* and the *Enterobacteriaceae* across studies underscores that positive association at the genus level cannot be equated with a promotive function.

The included studies did not agree on the relationship between diversity and infection. Higher diversity was reported in infected mosquitoes by (Bassene et al., 2018) but in uninfected mosquitoes by (Surat et al., 2016), while (Carlos et al., 2025) found reduced diversity in low-burden mosquitoes, and (Sandeu et al., 2025) found the direction to depend on season. Sharma (Sharma et al., 2020) added a temporal dimension, describing a transient decline in bacterial abundance during the early post-infection window. These differences are best attributed to heterogeneity among studies rather than to a single biological rule: the studies differ in mosquito species, geographic origin, parasite species, whether they contrasted infection status or oocyst burden, whether whole mosquitoes or dissected midguts were analyzed, and which sequencing platform and bioinformatic pipeline were used. The finding by (Bahia et al., 2014; Bassene et al., 2018) that larval breeding-site ecology, rather than infection, was the dominant driver of composition further cautions against interpreting diversity differences as infection-driven.

The evidence base is constrained by its methods. Most of the 13 studies relied on 16S rRNA amplicon sequencing rather than true shotgun metagenomics, which alone provides whole-community functional profiling and which no included study employed. Amplicon sequencing resolves most taxa only to the genus level, cannot reliably distinguish species or strains, and cannot distinguish live from dead bacteria; it is also subject to primer and hypervariable-region biases, which are compounded here by the mix of 454 and Illumina platforms and of operational taxonomic unit and amplicon sequence variant pipelines across studies. Critically, 16S data provide no direct measure of microbial function, so that the mechanisms inferred from taxonomic correlations in the observational studies remain largely speculative. The strain-level functional heterogeneity that best explains the *Serratia* paradox (Gao et al., 2021), is precisely what amplicon sequencing cannot detect. Functional and mechanistic validation was confined to a minority of studies (Bahia et al., 2014; Tchioffo et al., 2013; Feng et al., 2021; Gao et al., 2021), and multi-omics integration was rare: whole-genome sequencing appeared in two studies (Feng et al., 2021; Gao et al., 2021), transcriptomics in three (Carlos et al., 2025; Feng et al., 2021; Sharma et al., 2020), and metabolomics in one (Feng et al., 2021). The field would benefit from shotgun metagenomics to resolve strain-level and functional gene content, metatranscriptomics to measure microbial activity during infection, and metabolomics to identify bioactive metabolites mediating the effects reported here.

The identification of specific inhibitory bacteria provides candidate agents for transmission-blocking approaches: *Serratia marcescens* and *Serratia ureilytica* emerged as the strongest candidates, the latter shown by (Gao et al., 2021) to inhibit parasites through a defined secreted lipase and to disseminate through mosquito populations by vertical, trans-stadial, and venereal transmission. The demonstration that community depletion increases infection (Kalappa et al., 2018; Gendrin et al., 2015) supports the broader principle that a stable, intact microbiota reduces vector competence, and (Gendrin et al., 2015) additionally, antibiotics in ingested human blood were identified as a factor that can raise mosquito susceptibility, a consideration for settings with high antibiotic use. The consistent association of specific taxa with infection status also suggests that microbiome composition could serve as a marker for entomological surveillance. These implications should be advanced cautiously, since most rest on single-system experiments or on observational associations that have not yet been validated across vectors and settings.

### Future research priorities

The taxa identified as inhibitory in this review point to concrete, evidence-based opportunities for malaria control. The clearest example is the use of naturally protective bacteria as transmission-blocking agents, exemplified by *Serratia ureilytica*, which inhibits parasites through a defined secreted lipase and self-disseminates through mosquito populations (Gao et al., 2021). These findings connect directly to paratransgenesis, in which a naturally occurring mosquito symbiont is genetically modified to express and deliver antiplasmodial effector molecules within the vector (Cleanclay et al., 2026). In practice, symbiotic bacteria, fungi, or viruses are isolated from mosquito tissues or environmental reservoirs and cultured to pure strains (Wilke and Marrelli, 2015), after which genes encoding antiplasmodial peptides are introduced, often under blood-meal-inducible promoters, and the engineered symbionts recolonize the midgut when ingested in a sugar meal (Huang et al., 2022). Because these symbionts are transmitted vertically and horizontally, their antiparasitic effect can persist across successive generations (Huang et al., 2022). A clear methodological framework of paratransgenesis is well illustrated in **Supplementary Figure 3**. Symbionts, including *Asaia, Serratia, Pantoea*, and *Enterobacter* have been engineered to express effectors such as scorpine, EPIP, defensin, and SM1 peptides, with scorpine reported to inhibit P. falciparum oocysts by up to 97.8% (Cleanclay et al., 2026), and combined transgenesis and paratransgenesis approaches using midgut peptide MP2 and scorpine have achieved up to 89.8 per cent sporozoite inhibition (Wang et al., 2012). Alongside these active strategies, the finding that antibiotics in ingested human blood can raise mosquito susceptibility by disrupting the protective microbiota (Gendrin et al., 2015) identifies a transmission risk relevant to high-antibiotic-use settings and a corresponding surveillance consideration.

Realizing this potential will require the field to address four priorities that follow directly from the gaps mapped in this review. First, shotgun metagenomic and metatranscriptomic studies are needed to move beyond the genus-level taxonomy of current 16S rRNA surveys to strain-level and functional resolution, which is required to resolve the bidirectional behavior of Serratia and the Enterobacteriaceae. Second, the geographic and species base must be broadened, since the evidence is concentrated in West and Central Africa and Asia, with a single South American study and no representation of high-burden East Africa. Third, the many taxa that are so far only observationally associated with infection require experimental validation before any can be assigned a protective or promotive function. Together, these steps would convert the taxonomic associations mapped here into the mechanistic and functional understanding on which durable, microbiome-based vector-control strategies must be built.

### Limitations of this scoping review

This scoping review has several limitations. First, only 13 studies met the eligibility criteria despite a search spanning January 2000 to April 2026, reflecting the genuine recency and specificity of the intersection between molecular microbiome methods and mosquito-*Plasmodium* research rather than an overly restrictive strategy, as high-throughput studies of this system have appeared largely within the last decade. Second, the evidence is geographically concentrated, drawn mainly from West and Central Africa (Senegal, Cameroon) and Asia (India, Thailand, China), with a single study from South America and none from high-burden East Africa, which limits generalizability. Third, the included studies are heterogeneous in mosquito species (*An. gambiae*, *An. funestus*, *An. coluzzii*, *An. stephensi*, *An. minimus*, *An. darlingi*, and *An. sinensis*) and in *Plasmodium* species (*P. falciparum*, *P. vivax*, and *P. berghei*), which complicates direct comparison. Fourth, study designs varied widely, from cross-sectional field surveys to controlled infection experiments, and the majority were observational, so that many reported relationships are associations from which causality cannot be inferred. Fifth, the sequencing methodologies were heterogeneous, spanning 454 pyrosequencing and Illumina platforms and both operational taxonomic unit and amplicon sequence variant pipelines, which limits cross-study comparability. Sixth, the near-exclusive reliance on 16S rRNA amplicon profiling limited taxonomic resolution to the genus level in most cases and provided little functional information, so that the functional characterization of the microbiome remains limited and experimental validation of individual taxa is scarce.

## Conclusion

Drawing on 13 primary studies, this review indicates that the mosquito gut microbiome exerts a net inhibitory effect on *Plasmodium* development. The strongest evidence is experimental: depleting the microbiota increased infection, while challenge with specific isolates reduced it. Observational studies are consistent with this, reporting taxa such as *Burkholderia*, *Asaia*, *Sphingomonas*, *Pseudomonas*, and *Serratia* enriched in uninfected or lower-burden mosquitoes. The microbiota is not uniformly protective; however, the *Enterobacteriaceae*, *Serratia*, *Methylobacterium*, *Pantoea*, *Elizabethkingia* and *Chryseobacterium* were positively associated with infection. These positive associations are observational, and no study demonstrated a promotive effect experimentally.

Because the evidence rests predominantly on 16S rRNA profiling, which offers limited taxonomic and functional resolution, causality remains unresolved for many taxa, most notably the strain-dependent *Serratia* and *Enterobacteriaceae*. Advancing the field will require shotgun metagenomics, metatranscriptomics, and metabolomics, alongside longitudinal, geographically broader studies and experimental validation, to translate these associations into a mechanistic basis for sustainable, microbiome-based malaria vector control.

## Data Availability

The original contributions presented in the study are included in the article/**Supplementary Material**, further inquiries can be directed to the corresponding author/s.

## References

[B1] Adebayo A. H. Yakubu O. F. Popoola J. O. David L. C. Okenze G. N. Agbafor A. G. . (2018). Evaluation of antimalarial and biochemical profiles of Abaleria® in Plasmodium berghei-infected mice. Comp. Clin. Pathol. 27, 1595–1601. doi: 10.1007/s00580-018-2780-830311153

[B2] Aja-Macaya P. Conde-Pérez K. Trigo-Tasende N. Buetas E. Nasser-Ali M. Nión P. . (2025). Nanopore full length 16S rRNA gene sequencing increases species resolution in bacterial biomarker discovery. Sci. Rep. 15, 26486. doi: 10.1038/s41598-025-10999-8 40691262 PMC12280133

[B3] Arksey H. O’Malley L. (2005). Scoping studies: towards a methodological framework. Int. J. Soc. Res. Method. 8, 19–32. doi: 10.1080/1364557032000119616

[B4] Bahia A. C. Dong Y. Blumberg B. J. Mlambo G. Tripathi A. BenMarzouk-Hidalgo O. J. . (2014). Exploring Anopheles gut bacteria for Plasmodium blocking activity. Environ. Microbiol. 16, 2980–2994. doi: 10.1111/1462-2920.12381 PMC4099322 24428613

[B5] Bassene H. Niang E. H. A. Fenollar F. Dipankar B. Doucouré S. Ali E. . (2018). 16S metagenomic comparison of Plasmodium falciparum–infected and noninfected anopheles Gambiae and Anopheles funestus microbiota from Senegal. Am. J. Trop. Med. Hyg. 99, 1489–1498. doi: 10.4269/ajtmh.18-0263 30350766 PMC6283497

[B6] Boissière A. Tchioffo M. T. Bachar D. Abate L. Marie A. Nsango S. E. . (2012). Midgut microbiota of the malaria mosquito vector Anopheles Gambiae and interactions with Plasmodium falciparum infection. PloS Pathog. 8. doi: 10.1371/journal.ppat.1002742 22693451 PMC3364955

[B7] Carlos B. C. Voges K. Affonso P. H. D. A. Jaye A. Tong Rios C. Tinoco-Nunes B. . (2025). Metabolic reprogramming and gut microbiota ecology drive divergent Plasmodium vivax infection outcomes in Anopheles darlingi. PLoS Pathog. 21 (12), e1013823. doi: 10.1371/journal.ppat.1013823 PMC12758817 41433330

[B8] Charavanamuttu Y. Agyeman Wamba A. Taylor-Robinson A. W. . (2026). The evolving landscape of malaria prevention strategies: a review of recent developments. Pathogens 15, 137. doi: 10.3390/pathogens15020137 41754391 PMC12943406

[B9] Chinedu S. N. Bella-Omunagbe M. Okafor E. . (2024). Computational studies on 6-pyruvoyl tetrahydropterin synthase (6-PTPS) of Plasmodium falciparum. Bioinform. Biol. Insights 18, 11779322241230214. doi: 10.1177/11779322241230214 38333003 PMC10851736

[B10] Cleanclay W. D. Kernyuy F. B. Kintung I. F. Yensii N. G. Chick J. A. Obi A. M. M. . (2026). Evaluating paratransgenesis using engineered symbiotic bacteria for Plasmodium inhibition in mosquito vectors: a systematic review. PloS Negl.Trop. Dis. 20, e0013654. doi: 10.1371/journal.pntd.0013654 41678582 PMC12900360

[B11] Díaz S. Escobar J. S. Avila F. W. (2021). Identification and removal of potential contaminants in 16S rRNA gene sequence data sets from low-microbial-biomass samples: an example from mosquito tissues. mSphere 6, e00506-21. doi: 10.1128/mSphere.00506-21 PMC8265668 34133198

[B12] Feng Y. Peng Y. Wen H. Song X. An Y. Tang H. . (2021). Microbial tryptophan catabolism affects the vector competence of Anopheles. Microbiology. doi: 10.1101/2021.02.15.431262

[B13] Gao H. Bai L. Jiang Y. Huang W. Wang L. Li S. . (2021). A natural symbiotic bacterium drives mosquito refractoriness to Plasmodium infection via secretion of an antimalarial lipase. Nat. Microbiol. 6, 806–817. doi: 10.1038/s41564-021-00899-8 33958765 PMC9793891

[B14] Gao H. Cui C. Wang L. Jacobs-Lorena M. Wang S . (2020). Mosquito microbiota and implications for disease control. Trends Parasitol. 36, 98–111. doi: 10.1016/j.pt.2019.12.001 31866183 PMC9827750

[B15] Gendrin M. Rodgers F. H. Yerbanga R. S. Ouédraogo J. B. Basáñez M.-G. Cohuet A. . (2015). Antibiotics in ingested human blood affect the mosquito microbiota and capacity to transmit malaria. Nat. Commun. 6 (1), 5921. doi: 10.1038/ncomms6921 PMC4338536 25562286

[B16] Huang W. Vega-Rodriguez J. Kizito C. Cha S. J Jacobs-Lorena . (2022). Combining transgenesis with paratransgenesis to fight malaria. eLife 11, e77584. doi: 10.7554/elife.77584 36281969 PMC9596157

[B17] Johnson J. S. Spakowicz D. J. Hong B. Y. Petersen L. M. Demkowicz P. Chen L. . (2019). Evaluation of 16S rRNA gene sequencing for species and strain-level microbiome analysis. Nat. Commun. 10, 5029. doi: 10.1038/s41467-019-13036-1 31695033 PMC6834636

[B18] Kalappa D. M. Subramani P. A. Basavanna S. K. Ghosh S. K. Sundaramurthy V. Uragayala S. . (2018). Influence of midgut microbiota in Anopheles stephensi on Plasmodium berghei infections. Malar. J. 17. doi: 10.1186/s12936-018-2535-7 30359252 PMC6203276

[B19] Liu H. Yin J. Huang X. Zang C. Zhang Y. Cao J. . (2024). Mosquito gut microbiota: a review. Pathogens 13, 691. doi: 10.3390/pathogens13080691 39204291 PMC11357333

[B20] Naandam S. M. Chataa P. Gogovi G. K. (2025). Analysis of a malaria transmission model with vaccination proportion and vaccine-induced immunity. MCA 30, 15. doi: 10.3390/mca3001001530654563

[B21] Nwinyi O. C. Balogun D. O. Isibor P. O. Prevalence and insecticide resistance in Anopheles coluzzii in Ado-Ota, Nigeria. doi: 10.1099/acmi.0.001002.v1

[B22] Oladipo H. J. Tajudeen Y. A. Oladunjoye I. O . (2022). Increasing challenges of malaria control in sub-Saharan Africa: priorities for public health research and policymakers. Ann. Med. Surg. 81. doi: 10.1016/j.amsu.2022.104366 36046715 PMC9421173

[B23] Ortiz‐Estrada ÁM. Gollas‐Galván T. Martınez Córdova L. R . (2019). Predictive functional profiles using metagenomic 16S rRNA data: a novel approach to understanding the microbial ecology of aquaculture systems. Rev. Aquacult. 11, 234–245. doi: 10.1111/raq.1223740046247

[B24] Romoli O. Gendrin M. (2018). The tripartite interactions between the mosquito, its microbiota and Plasmodium. Parasites Vectors 11. doi: 10.1186/s13071-018-2784-x 29558973 PMC5861617

[B25] Sandeu M. M. Tatsinkou C. G. M. Dada N. Feukam F. K. Njiokou F. Hughes G. L. . (2025). Microbiota diversity of Anopheles Gambiae in Bankeng, southern Cameroon, and its association with Plasmodium falciparum infection. mSphere 10, 1–16. doi: 10.1128/msphere.00490-25 PMC12724382 41347760

[B26] Sharma P. Rani J. Chauhan C. Kumari S. Tevatiya S. Das De T. . (2020). Altered gut microbiota and immunity defines Plasmodium vivax survival in Anopheles stephensi. Front. Immunol. 11. doi: 10.3389/fimmu.2020.00609 32477320 PMC7240202

[B27] Sharpton T. J. (2014). An introduction to the analysis of shotgun metagenomic data. Front. Plant Sci. 5. doi: 10.3389/fpls.2014.00209 24982662 PMC4059276

[B28] Su X. Zhang C. Joy D. A. (2020). Host-malaria parasite interactions and impacts on mutual evolution. Front. Cell. Infect. Microbiol. 10, 587933. doi: 10.3389/fcimb.2020.587933 33194831 PMC7652737

[B29] Surat W. Mhuantong W. Sangsrakru D. Chareonviriyaphap T. Arunyawat U. Kubera A. . (2016). Gut bacterial diversity in plasmodium-infected and plasmodium-uninfected anopheles minimus. Chiang Mai J. Sci. 43, 426–439.

[B30] Tchioffo M. T. Boissière A. Abate L. Nsango S. E. Bayibéki A. N. Awono-Ambéné P. H. . (2016). Dynamics of bacterial community composition in the malaria mosquito’s epithelia. Front. Microbiol. 6. doi: 10.3389/fmicb.2015.01500 26779155 PMC4700937

[B31] Tchioffo M. T. Boissière A. Churcher T. S. Abate L. Gimonneau G. Nsango S. E. . (2013). Modulation of malaria infection in Anopheles Gambiae mosquitoes exposed to natural midgut bacteria. PloS One 8. doi: 10.1371/journal.pone.0081663 24324714 PMC3855763

[B32] Tebamifor M. E. Cleanclay W. D. Mamudu C. O. Ogunlana O. O . (2025). Surveillance of Wolbachia infection in mosquito species in Ota, Ogun State, Nigeria. Discov. Appl. Sci. 7, 270. doi: 10.1007/s42452-025-06686-y30311153

[B33] Tiwari N. Bansal M. Santhiya D. Sharma J. G . (2022). Insights into microbial diversity on plastisphere by multi-omics. Arch. Microbiol. 204, 216. doi: 10.1007/s00203-022-02806-z 35316402

[B34] Tricco A. C. Lillie E. Zarin W. O’Brien K. K. Colquhoun H. Levac D. . (2018). PRISMA extension for scoping reviews (PRISMA-ScR): checklist and explanation. Ann. Intern. Med. 169, 467–473. doi: 10.7326/m18-0850 30178033

[B35] Varo R. Chaccour C. Bassat Q. (2020). Update on malaria. Med. Clínica 155, 395–402. doi: 10.1016/j.medcle.2020.05.024 32620355

[B36] Wakai T. Fiamitia C. Kintung I. Johngwe M. Chinedu S. Afolabi I. (2025). Knowledge, practices, and perceptions towards malaria prevention and control among residents of Canaanland and surrounding areas in Ota, Ogun State, Nigeria: a cross-sectional study. Front. Trop. Dis. 6, 1686197. doi: 10.3389/fitd.2025.1686197

[B37] Wakai T. N. Yensii N. G. Kernyuy F. B. Bella-Omunagbe M. Chinedu S. N. Afolabi I. S. (2026). Global research landscape of telomere biology in infectious diseases: mechanistic links between host–pathogen interactions and immune ageing. Front. Aging 6, 1729868. doi: 10.3389/fragi.2025.1729868 PMC12823901 41585361

[B38] Wang S. Ghosh A. K. Bongio N. Stebbings K. A. Lampe D. J. Jacobs-Lorena M. (2012). Fighting malaria with engineered symbiotic bacteria from vector mosquitoes. Proc. Natl. Acad. Sci. U.S.A. 109 (31), 12734–12739. doi: 10.1073/pnas.1204158109 PMC3412027 22802646

[B39] Wilke A. B. B. Marrelli M. T. (2015). Paratransgenesis: a promising new strategy for mosquito vector control. Parasites Vectors 8. doi: 10.1186/s13071-015-0959-2 26104575 PMC4489152

[B40] World Malaria Report 2025. Available online at: https://www.who.int/teams/global-malaria-programme/reports/world-malaria-report-2025 (Accessed December 24, 2025).

[B41] Yensii N. G. Kernyuy F. B. Wakai T. N. Dokunmu T. M. Ogunlana O. O. (2026). Bibliometric trends and emerging frontiers in RNA interference research for mosquito control (2010–2025). Front. Insect Sci. 6, 1758530. doi: 10.3389/finsc.2026.1758530 PMC13036116 41924196

[B42] Zheng R. Wang Q. Wu R. Paradkar P. N. Hoffmann A. A. Wang G.-H. (2023). Holobiont perspectives on tripartite interactions among microbiota, mosquitoes, and pathogens. ISME J. 17 (8), 1143–1152. doi: 10.1038/s41396-023-01436-7 PMC10356850 37231184

