# Supplementary material for: The mosquito gut microbiome and Plasmodium development: a scoping review of molecular approaches and implications for malaria vector control
Source: Front Cell Infect Microbiol. 2026 Sep 30;16:1906816. doi: 10.3389/fcimb.2026.1906816 (PMC13661432; doi:10.3389/fcimb.2026.1906816)
Supplement: Supplementary file 1 [file Supplementaryfile1.docx]

**SUPPLEMENTARY FIGURES**

The following figures illustrate general 16S rRNA and shotgun metagenomic sequencing workflows referred to in the Introduction. They describe standard laboratory methodology rather than review findings and are provided here for reference.

**Supplementary Figure S1:** The process of 16S rRNA sequencing from sample collection to final analysis. Figure by F.B Kernyuy, and designed with Canva

**Supplementary Figure S2:** Shotgun Metagenomics pipeline from sample collection to Analysis. Figure by F.B Kernyuy, and designed with Canva

**Supplementary Figure S3:** Genetic-Based approach for controlling vector-borne diseases. Figure by F.B Kernyuy, designed with Canva
